# Big data simulations for capacity improvement in a general ophthalmology clinic

**DOI:** 10.1007/s00417-020-05040-9

**Published:** 2021-01-02

**Authors:** Christoph Kern, André König, Dun Jack Fu, Benedikt Schworm, Armin Wolf, Siegfried Priglinger, Karsten U. Kortuem

**Affiliations:** 1 Department of Ophthalmology, University Hospital LMU Munich, Mathildenstraße 8, 80336 Munich, Germany; 2 Moorfields Eye Hospital, London, UK; 3 Department of Ophthalmology, Ulm University, Ulm, Germany

**Keywords:** Ophthalmology, Waiting time optimisation, Clinic efficiency, Discrete event simulation, Big data

## Abstract

**Purpose:**

Long total waiting times (TWT) experienced by patients during a clinic visit have a significant adverse effect on patient’s satisfaction. Our aim was to use big data simulations of a patient scheduling calendar and its effect on TWT in a general ophthalmology clinic. Based on the simulation, we implemented changes to the calendar and verified their effect on TWT in clinical practice.

**Design and methods:**

For this retrospective simulation study, we generated a discrete event simulation (DES) model based on clinical timepoints of 4.401 visits to our clinic. All data points were exported from our clinical warehouse for further processing. If not available from the electronic health record, manual time measurements of the process were used. Various patient scheduling models were simulated and evaluated based on their reduction of TWT. The most promising model was implemented into clinical practice in 2017.

**Results:**

During validation of our simulation model, we achieved a high agreement of mean TWT between the real data (229 ± 100 min) and the corresponding simulated data (225 ± 112 min). This indicates a high quality of the simulation model. Following the simulations, a patient scheduling calendar was introduced, which, compared with the old calendar, provided block intervals and extended time windows for patients. The simulated TWT of this model was 153 min. After implementation in clinical practice, TWT per patient in our general ophthalmology clinic has been reduced from 229 ± 100 to 183 ± 89 min.

**Conclusion:**

By implementing a big data simulation model, we have achieved a cost-neutral reduction of the mean TWT by 21%. Big data simulation enables users to evaluate variations to an existing system before implementation into clinical practice. Various models for improving patient flow or reducing capacity loads can be evaluated cost-effectively.

**Supplementary Information:**

The online version contains supplementary material available at 10.1007/s00417-020-05040-9.

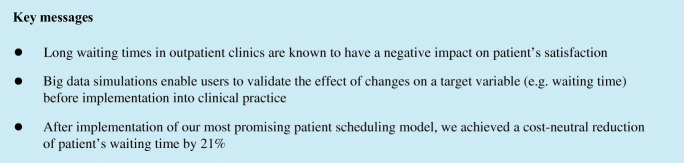

## Introduction

Modern healthcare systems struggle to provide high-quality health services for the population because of limited capacity and resources and concomitantly face an increasing number of patients attributable to demographic changes. [1] Budget restrictions emphasise the importance of economic evaluations in the healthcare sector to ensure the efficient and effective use of available resources [2]. Moreover, patient online reviews play an increasingly important role in a patient’s choice of healthcare provider [3]. Friendliness, empathy, cost, and especially time spent with patients and waiting times are given as being important factors in online reviews [4]. Waiting times have been shown to be a major cause for patient satisfaction in multiple clinical studies [5–8]. The reported correlation between waiting time and patients’ satisfaction directly influences the perceived quality of healthcare [9]. Quality management without waiting time optimisation is therefore inconceivable.

The pioneers of patient scheduling optimisation, Welch and Bailey, stated in the early 1950s that the ideal workload of the clinic can be achieved by scheduling patients at fixed intervals [10]. By basing their assumptions on the fact that a doctor’s working time is more precious than a patient’s time, they were willing to accept long waiting times for patients in clinics. Newer studies suggest that patient scheduling should aim at improving patients’ satisfaction by waiting time reduction rather than be based on the ideal occupancy rate of the resource “doctor” [11]. Discrete event simulation (DES) is a method for developing and testing operational solutions over time prior to their implementation in an operating system [12]. This offers a cost-effective solution for imitating and therefore for evaluating the effect of changes on an existing real-world system.

In this study, DES was used to analyse the effect of various patient appointment scheduling models in a general ophthalmology clinic at a tertiary referral centre. Their effect on actual patient flow and their waiting times were examined prior to implementation of the most promising model into clinical practice. Patients’ waiting time after implementation is reported as a marker for improved patient flow and resolves capacity strains in clinic.

## Methods

### Study setting and design

This retrospective simulation study performed at the University Eye Hospital of the Ludwig-Maximilian University, Munich, Germany, was designed (i) to create a simulation model on patient flow in our general ophthalmology clinic; (ii) to simulate different patient appointment schedules; and (iii) to implement the best model into clinical practice. Ethical approval for the study was obtained from the Institutional Review Board of the University Eye Hospital Munich in Germany. The study adheres to the Declaration of Helsinki. All data warehouse queries were approved by the local data protection officer.

### Study outcomes

Primary outcome measurement was the reduction in patients’ mean “total waiting time” (TWT) after implementation of an advantageous patient scheduling model derived from the simulation analysis. TWT was defined as the time between two timepoints: (i) the patient’s registration at the admission desk and (ii) the last editing of the patient’s electronic health record (EHR) by an ophthalmologist. Secondary outcome measures were the real state of waiting and process times, the implementation of the DES model and its validation, and the simulation of the effect of the different patient appointment scheduling models on the TWT.

### Data source

Administrative information and timepoints used for further processing were extracted from a data warehouse in use since 2012 [13]. This warehouse contains clinical findings from the patients’ EHR; a customized version of i.s.h. med (Cerner AG, Erlangen, Germany) and investigation results for more than 393,000 patients as of February 2020 [14]. Moreover, all digital movements of the patients in the clinic are recorded with a timestamp (start of registration, consultation, and diagnostics). All timepoints are exported as a Microsoft Excel sheet (Microsoft, Richmond, USA) for further processing. Process times that could not be extracted from the database were measured manually, e.g. anterior segment examination or fundoscopy. For the simulation process, digital movements including timestamps for all patients in our general ophthalmology clinic between January and December 2014 were exported as metadata. To measure the effect on TWT after implementation of one of the simulated models, another metadata set was created between October 2016 and June 2017.

### Data processing and simulation

An operational research technique, namely, discrete event simulation (DES), was used to assess the efficacy of the current patient flow and to forecast the effect of modality changes as appointment times or examinations on patient flow without alterations to the present system [15, 16]. The model was created by using a simulation and modelling software customised for healthcare process analysis (FlexSim Healthcare 3D, Version 5.0.2, FlexSim Software Products Inc., Orem, Utah, USA). The minimal number of replications of the DES model was calculated with Stat::Fit (Geer Mountain Software Corp., South Kent, USA). The following major steps are necessary in the process of conducting a simulation study of any discrete system and are presented in the “Results” part of this manuscript: (i) generation of a simulation model by patient flow analysis, process time measurements, and validation of the model; (ii) experimentation (examination of the effect of modality changes in the patients’ appointment schedules on the TWT); and (iii) implementation in clinical practice. [17]

### Statistics

All clinical data used for this study were queried from the data warehouse as an Excel file and exported to IBM SPSS Statistics for Windows, Version 25.0 (IBM Corp. Armonk, USA), for further statistical analyses. Deviation of metadata and simulated data was assessed by the Kolmogorov-Smirnov test for normality. As data were not normally distributed, paired differences in distribution between meta- and simulated data were analysed by the Mann-Whitney *U* test to validate our simulation model. Results are presented as mean values including standard deviation (mean ± SD). The level of statistical significance was set at 0.05. The maximal range of estimates for the DES model was defined as 15 min at a confidence level of 95%.

## Results

### Patient flow analysis

**Fig. 1:**
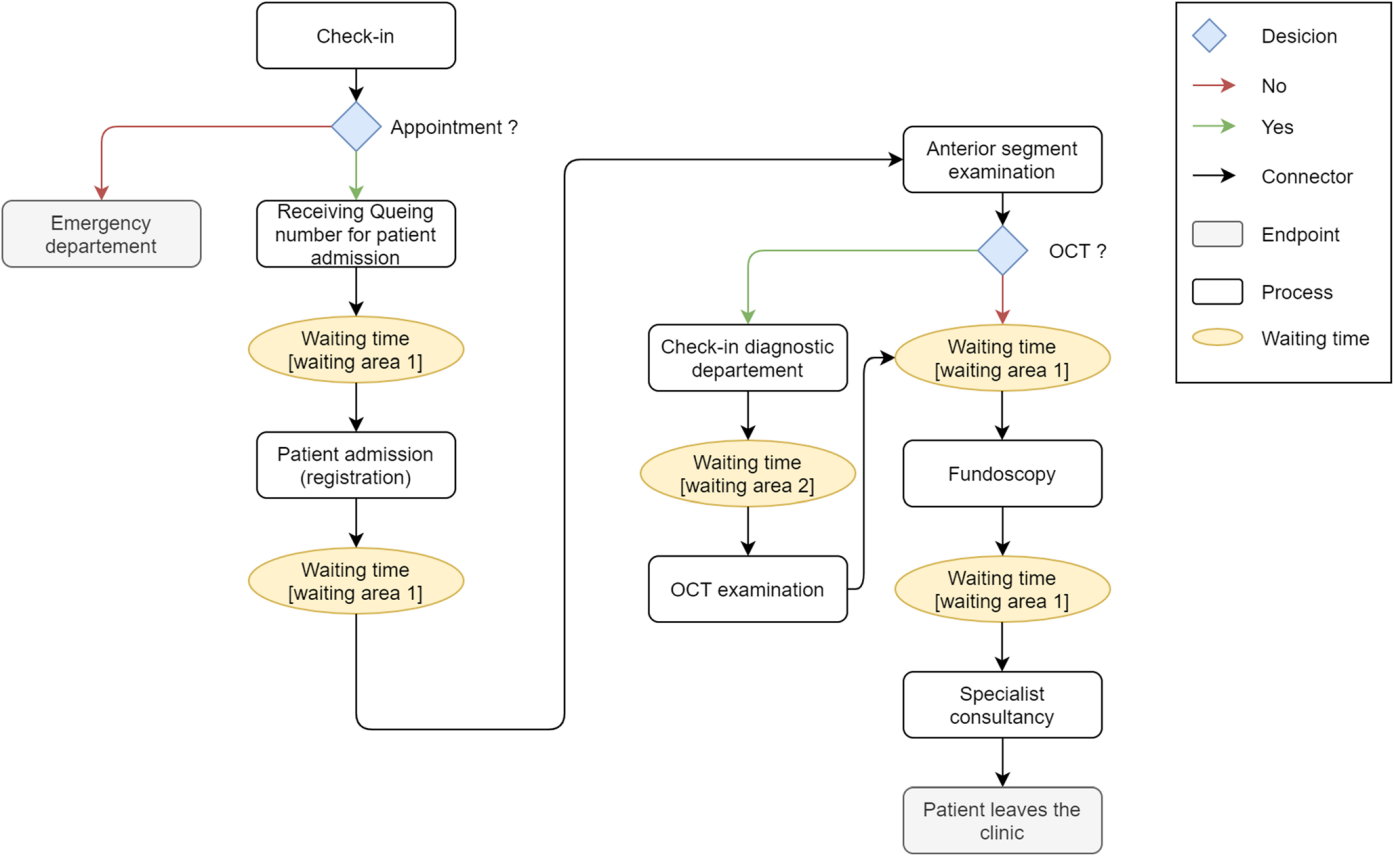
Patient flow diagram. This represents the pathway including waiting times of scheduled general ophthalmology patients from check-in, through examination (± OCT scan) to consultant presentation before leaving the clinic

The first step in DES comprises patient flow analysis. All patients arriving in the hospital are triaged as being an emergency or as having scheduled appointments. For this study, we assessed scheduled patients for the general ophthalmology service. After registration, all patients undergo an anterior segment examination including visual acuity and intraocular pressure measurements by a resident, followed by the application of dilating eye-drops. Depending on medical necessity, an OCT scan is performed prior to binocular fundoscopy. Before leaving the clinic, patients are presented to a consultant for final evaluation (Fig. 1). To improve clarity for the reader, a video of our patient pathway simulation in FlexSim can be found in the supplemental material (supplemental video 1).

### Current patients’ appointment schedule

The actual state of the patient scheduling calendar in our clinic consisted of admission times between 7:30 am and 12:50 pm. Until 11:50 am, patients were scheduled in 10-min slots. Follow-up and new referrals were randomly mixed. At 12:20, 12:40, and 12:50 pm, three follow-up patients were scheduled for the same slot (“triple” appointment). The 7:30 am appointment was a “double” appointment where the first 2 patients of the day arrived at the same time. Staff included two residents and one consultant. The maximum number of patients scheduled per day and resident was 25 (50 in total).

### Waiting time analysis

Only digitally available timepoints could be exported on a large scale to generate a simulation model. These were (i) registration at admission; (ii) first contact with the doctor during anterior segment examination; (iii) undergoing optical coherence tomography (OCT) scan; and (iv) end of examination after specialist consultancy. In total, timepoints of 4.401 visits in our general ophthalmology clinic were selected between January and December 2014, of which 33% (1.452 visits) received an OCT scan and 43% (1.892 visits) were first referrals. On average, 25 patients per resident attended the clinic during working days. Mean TWT on the day visit was 229 ± 100 min for all patients. The following waiting times could be stratified per subgroup: 234 min for patients undergoing an OCT scan, 237 min for new referrals, and 223 min for follow-up visits. On average, patients spent 101 min in the waiting area before first contact with the doctor. Time between anterior segment examination and receiving an OCT scan was 28 min.

As mentioned above, not all timepoints could be exported from the warehouse. Manual time measurements were performed for the following processes: registration, anterior segment examination, fundoscopy, OCT examination, and specialist consultancy, including their ideal distribution in the DES model (Table 1).

**Table 1:** Manual measurements of process times in our outpatient clinic

Process step	Mean ± SD (minutes)	Number of measurements (*n*)	Distribution of parameters
Registration	3 ± 1	25	Log-Laplace
Anterior segment examination	20 ± 8	23	Johnson bounded
New referrals	24 ± 7	15	Beta
Follow-up visits	12 ± 3	20	Weibull
Binocular fundoscopy	7 ± 2	49	Beta
OCT examination	4 ± 1	25	Johnson bounded
Specialist consultancy	8 ± 5	23	Johnson bounded

### Generating and validating the simulation model

The DES model was created based on patient flow, metadata previously exported from the data warehouse, and manually measured process times. The definition of the minimal number of replications of a simulation model is necessary to guarantee the precision of the predictions based on the previously defined maximal range of estimates of 15 min at a confidence level of 95%. The minimal number of replications of our current patient appointment calendar, based on metadata from the warehouse, was 302. Depending on the simulated scenario (current state and Model 1 to 9—see Table 3), between 224 and 399 replications of the model were necessary to reach our maximum range for estimates of 15 min on a 95% confidence level. To be on the safe side for all models, we were planning to simulate at a later stage; the minimal number of replications during the experiments was set to 500. To validate the model, it was necessary to compare the distribution of means between not normally distributed metadata and simulated data by the Mann-Whitney *U* test. The simulation model was adapted manually until no difference was found between the distribution of means of total waiting time, which implicates comparability between metadata and simulated data (*p* value = 0.72). The simulation model precisely reproduced process times that were generated from the metadata as shown in Table 2. Comparability of means between metadata and our DES model implies a realistic representation of the actual patient flow in our clinic. [18]

**Table 2:** Validating simulated data to metadata from the clinical warehouse

	Metadata	Simulated data
Total number of patients (n)	4.401	9.000
TWT (min)	229 ± 100	225 ± 112
Test for normal deviation *	*≤ 0.01*	*≤ 0.01*
Distribution of means **	*0.72*	
Patients per day (*n*)	18	18
New referrals	6 (33%)	6 (33%)
Needing OCT scan	8 (44%)	8 (44%)
TWT new referrals (min)	237 ± 103	227
TWT for OCT scan (min)	235 ± 97	247
Waiting time registration to examination (min)	101 ± 65	85
Time from examination to OCT scan (min)	29 ± 38	23

*Kolmogorov-Smirnov test for normal deviation (*p* ≤ 0.05 implies non-normally deviated data)

**Mann-Whitney *U* test to compare distribution of means between the groups metadata and simulated data (paired differences test)

### Simulation of various patient appointment schedules

We examined the effects of changes in the patient appointment scheduling calendar on the TWT of patients in the outpatient department by using the previously validated DES model. In addition to the originally validated DES model (no changes = current state), we examined the effect of nine different changes in the patients’ admission schedule and staffing on total waiting time (Table 3). Our simulated patients’ admission schedules are based on published models, namely, block intervals (model 4) (a certain number of patients arrive at the same time) [19]; fixed intervals (model 5) (varying by number of minutes per slot) [20]; a combination of both with “double” or “triple” appointments at the beginning of each session, followed by fixed intervals (models 2 and 3) [21]. Further alterations to the scheduling calendar, as described in the literature, were simulated: appointments with longest TWT were cancelled and rebooked in fixed intervals at the end of the day (model 1); staffing was increased (models 6, 8, and 9); the order of new referral and follow-ups was changed (model 7) [22, 23].

**Table 3:** Overview of scheduling models including the current state and nine different simulated models

Model	Slot per patient (min)	Simulated patients per resident (n)	Number of residents and consultants (*n*; *n*)	Changes compared to the current state	Simulated TWT (mean)
Current state	10	18	2; 1	As explained in the “Results” part “currents patients’ appointment schedule”	225
1	10	18	2; 1	Cancellation of three appointments with longest TWT. Cancelled appointments were scheduled after 12:50 pm in 10-min intervals	178
2	20	19	2; 1	“Triple” appointments at 7:30 and 10:00 am. Fixed 20-min intervals from 8 am to 2 pm	153
3	15	18	2; 1	“Triple” appointments at 8:00 am; “double appointments” at 10 am and 1 pm, fixed 15-min intervals from 8 am to 2 pm	181
4	15	18	2; 1	Fixed block intervals: four patients every hour at the same time from 8 am to 2 pm	164
5	10	18	2; 1	Model 1 without the 7:30 am “double” appointment. Fixed 10-min intervals from 7:30 am to 12:50 pm	169
6	10	22	3; 1	Current state with 1 additional resident	214
7	10	17	2; 1	Current state but new patients were scheduled prior to follow-up patients.	238
8	10	23	3; 2	Current state with 1 additional resident and consultant (increased staffing)	216
9	10	22	3; 1	Model 2 with 1 additional resident (increased staffing)	125

### Implementation into clinical practice

**Fig. 2:**
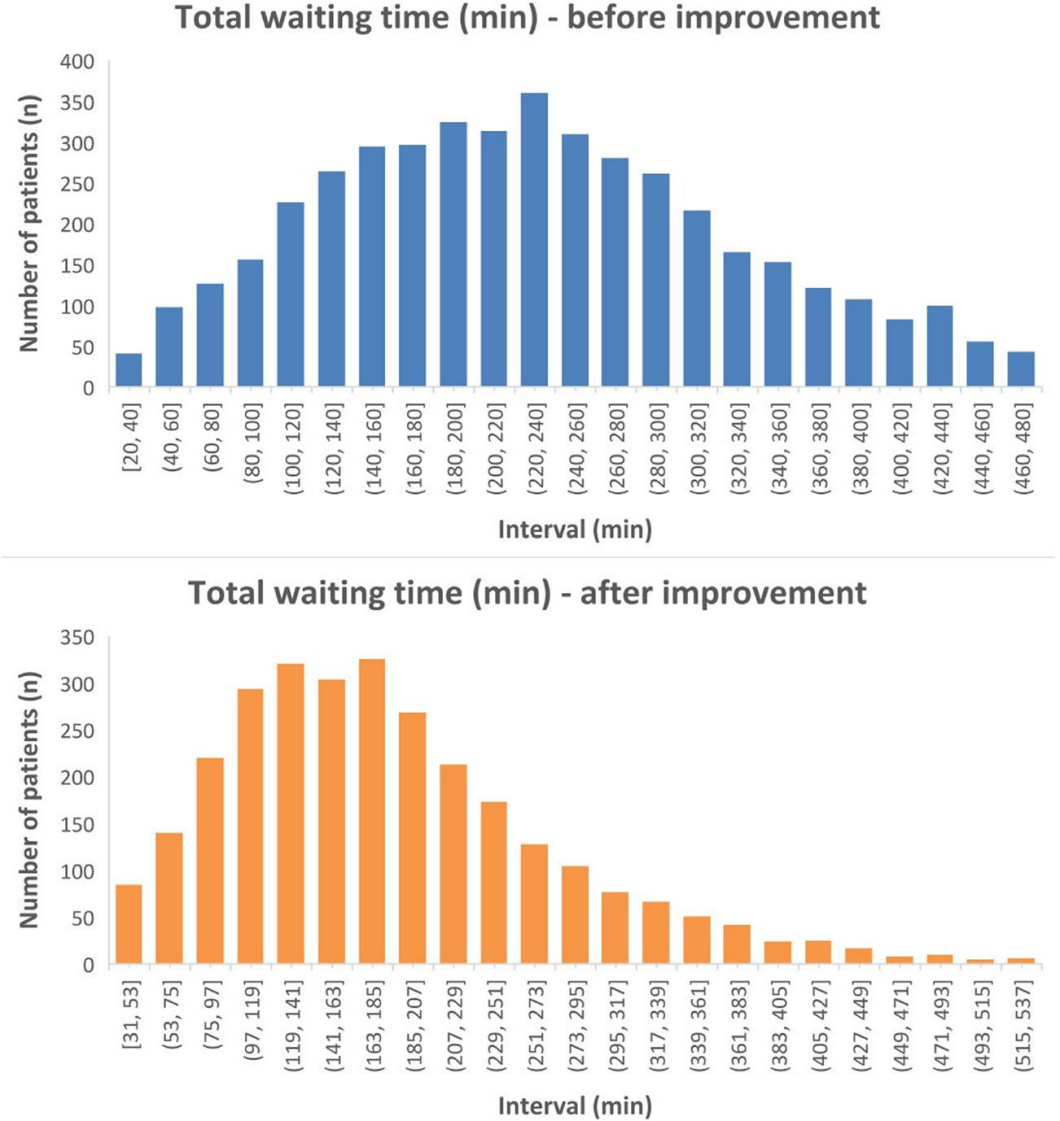
Histogram analysis of TWT before and after implementation of model 2. Intervals on x-axis are set to 20 min

Model 2 was implemented into clinical practice in 2016. This model resulted in the maximal reduction of total waiting time, without increasing the number of clinic staff. After 9 months of operation, another metadata set was exported in 2017 and compared with the initial state before the performance of big data simulations. This dataset consists of 2.909 visits. Mean total waiting time was reduced from 229 ± 100 min before to 183 ± 89 min after its implementation in 2017 (Figure 2).

## Discussion

### Main findings

Total waiting time of patients in our general ophthalmology outpatient department has been reduced by 21% from 229 ± 100 to 183 ± 89 min. This was achieved by big data simulations based on a discrete event simulation model. Various changes in patient scheduling were compared easily and cost-effectively by their effect on waiting times in clinical practice. This reduction in waiting time was achieved without the hiring of new staff or the acquisition of new machines or reconstruction work and resulted in a capacity improvement in our clinic.

### Findings in the light of the current literature

To our knowledge, this is the first study to use data derived from a clinical data warehouse for process and waiting time optimisation. In countries with highly developed healthcare systems, approximately 80% of small and general hospitals employ EHR [24]. As of 2019, only one-third of them truly work in a paperless fashion leaving a huge gap for further digitalisation. This progress will make data, stored in corresponding data warehouses, more and more accessible and will allow the application of comparable big data simulations as presented in this study on a broader scale, thereby affecting quality management and relieving capacity strains in healthcare [25].

DES models have previously been used for improving patient appointment scheduling. Waiting time of patients has been reduced by the wider distribution of appointment slots and the rearrangement of new and follow-up slots [18]. These results correspond to ours: increasing the slot time per patient from 10 (current state) to 15 (model 4) and 20 min (model 2) results in a simulated reduction of TWT from 229 min to 164 and 153 min, respectively. On the contrary, they reach process optimisation by reserving afternoon slots exclusively for follow-up patients with similar pathways. In our DES model, this approach even increased TWT as compared with the initial state from 229 to 238 min. This discrepancy might be explained by the training grade of staff in our general ophthalmology clinic, namely, “year one” residents without knowledge of common pathways for follow-up patients.

Another approach focused on the reduction of patient visit times and originates from industrial manufacture: the Lean Six Sigma technique maximises positive results, namely, the reduction of patient waiting time [26]. After implementing the most promising model into clinical practice at a vitreoretinal outpatient clinic, patient visit times were reduced by 18%. Our model achieved a comparable reduction of TWT of 21%.

Multiple studies have shown that up to 80% of patients arrive prior to their actual appointment [27, 28]. In addition to patient appointment schedule optimisation, appointment discipline might improve waiting times in clinics. Low appointment discipline results not only in waiting area congestion but also in a suboptimal use of available resources because of the irregular arrival of patients [29].

### Strengths and limitations

The major advantage of DES models is that changes in patient scheduling can be implemented easily and cost-effectively into clinical practice without the hiring of new staff or the acquisition of new machines or reconstruction work [12]. Moreover, DES allows the reduction of patient waiting time, resource overtime, and waiting area congestion by the simulation and optimisation of the use of available resources. [29]

On the other hand, the accuracy of our DES model might be compromised by the following circumstance. Only timestamps from the EHR and manual process time measurements could be used for generating the model. The first timestamp available is the first contact with our administration office. The initial waiting time of the patient after receiving the queuing number is therefore not represented in the model (Fig. 1). Two reasons are responsible for this fact: (i) the queuing number machine is not connected to the data warehouse or the EHR; (ii) patients with appointments in our general ophthalmology clinic cannot be identified upon arrival in the clinic prior to their check-in at the admission desk. Measurement of the initial waiting time was therefore not possible.

### Implications for further research

As discussed previously, waiting time (negative correlation) and consultation time (positive correlation) are correlated with patient satisfaction. This correlation is significantly stronger in the first 90 min of the waiting time and in the first 15 min of the consultation time [30]. On the other hand, a waiting time of up to 45 min has no impact on patient satisfaction and seems to be acceptable [8]. Manual process measurements in this study have shown that patients spent in total 39 min with a doctor during their visit (including anterior segment and fundus examination, OCT, and specialist consultancy). This leaves 146 min of real waiting time resulting in a strong negative impact on patient satisfaction. Future studies should include patient satisfaction in big data simulation models.

### Implication for clinical practice

We believe that DES simulations, as presented in this study, might be applicable in other settings such as at private ophthalmologist or other healthcare systems. The private sector is facing an increasing number of patients and waiting times, whilst competing for valuable patients. Because waiting time has a direct correlation on patient satisfaction and therefore recommendation rates, DES might be an economically valuable tool outside larger ophthalmic referral centres [3, 4, 25]. In the light of digitalization of healthcare and therefore the broader availability of metadata, this method can be used to optimize patient flow and therefore waiting time without producing additional costs. By its cost-effectiveness and adaptability to other settings, this method is qualified for implementation in other healthcare systems with limited resources as well.

### Conclusion

Big data simulations offer the opportunity to simulate patient appointment scheduling cost-effectively and efficiently but without introducing alterations to an existing system. After implementation of the most promising simulation model, patients’ total waiting time in our outpatient clinic could be reduced by 46 min (− 21%). Simulation models might thus improve patient flow and capacity strains in other ophthalmologic services.

## Supplementary Information

Supplemental Video 1 Simulation model in FlexSim of a regular Monday general ophthalmology clinic from 7:30 am to 4:30 pm. Comments on the video: 00:00–00:16 Simulation of the first patient of the day. The patient enters the hospital at 7:30 am. In the right upper corner, you can see the simulated time of the day. Right click allows changes in perspective. Left click lets the user move through the model. Running speed can be changed at the top. 00:16–00:45 The patient enters the waiting area in front of the admission desk. Here, we are simulating a patient without OCT scan. 00:45–01:50 The patient passes through the next steps of our patient’s pathway (see Fig. 1). The resource doctor is available from 8 am. Another patient needing an OCT scan enters the model at 01:20. 01:50–03:50 More and more patients enter the clinic during business hours from 7:30 am to 4:30 pm. Right clicking on the patient allows the user to visualize the actual step of our simulated patient’s pathway (e.g. admission, waiting area, anterior segment, examination). 03:18–03:43 First results of patients who finished the day are available. The program presents the statistic output to the user as follows: number of patients; average waiting time of different milestones; total waiting time stratified for all patients, OCT, new referrals, and follow-up patients. Results can be exported to an Excel sheet for further processing. 03:44 All patients finished their day in our simulated clinic. (AVI 11033 kb)
